# Supplementary figures and images for: Disc and Actin Associated Protein 1 influences attachment in the intestinal parasite Giardia lamblia
Source: PLoS Pathog. 2022 Mar 25;18(3):e1010433. doi: 10.1371/journal.ppat.1010433 (PMC8986099; doi:10.1371/journal.ppat.1010433)

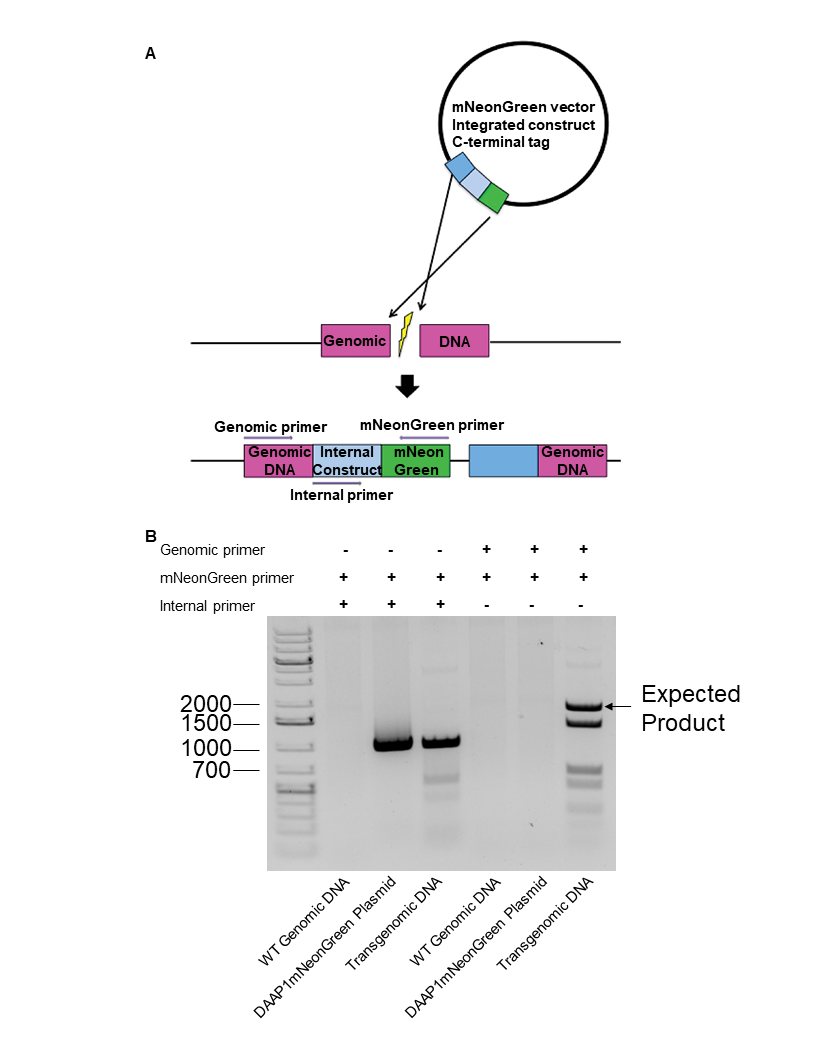

Supplement: S5 Fig — A) Schematic for integration and position of PCR primers used to verify integration. B) Image of agarose gel with PCR products confirming genomic integration of DAAP1-mNeonGreen. (PNG) [file ppat.1010433.s005.png]
